# Implementing the “Best Template Searching” tool into Adenosiland platform

**DOI:** 10.1186/2193-9616-1-25

**Published:** 2013-12-20

**Authors:** Matteo Floris, Davide Sabbadin, Antonella Ciancetta, Ricardo Medda, Alberto Cuzzolin, Stefano Moro

**Affiliations:** CRS4, Parco Polaris, 09010 Pula, CA Italy; Molecular Modeling Section (MMS), Department of Pharmaceutical and Pharmacological Sciences, University of Padova, via Marzolo 5, I-35131 Padova, Italy

**Keywords:** G protein-coupled receptors, Adenosine receptors, Receptor modelling, Bioinformatics platform, Adenosiland

## Abstract

**Background:**

Adenosine receptors (ARs) belong to the G protein-coupled receptors (GCPRs) family. The recent release of X-ray structures of the human A_2A_ AR (h A_2A_ AR ) in complex with agonists and antagonists has increased the application of structure-based drug design approaches to this class of receptors. Among them, homology modeling represents the method of choice to gather structural information on the other receptor subtypes, namely A_1_, A_2B_, and A_3_ ARs. With the aim of helping users in the selection of either a template to build its own models or ARs homology models publicly available on our platform, we implemented our web-resource dedicated to ARs, *Adenosiland*, with the “*Best Template Searching*” facility. This tool is freely accessible at the following web address: http://mms.dsfarm.unipd.it/Adenosiland/ligand.php.

**Findings:**

The template suggestions and homology models provided by the “*Best Template Searching*” tool are guided by the similarity of a query structure (putative or known ARs ligand) with all ligands co-crystallized with hA_2A_ AR subtype. The tool computes several similarity indexes and sort the outcoming results according to the index selected by the user.

**Conclusions:**

We have implemented our web-resource dedicated to ARs *Adenosiland* with the “*Best Template Searching*” facility, a tool to guide template and models selection for hARs modelling. The underlying idea of our new facility, that is the selection of a template (or models built upon a template) whose co-crystallized ligand shares the highest similarity with the query structure, can be easily extended to other GPCRs.

## Findings

The template suggestions and homology models provided by the “*Best Template Searching*”tool are guided by the similarity of a query structure (putative or known ARs ligand) with all ligands co-crystallized with hA_2A_ AR subtype. The tool computes several similarity indexes and sort the outcoming results according to the index selected by the user.

### Background

Adenosine receptors (ARs) belong to the G protein-coupled receptors (GCPRs) family. The known four subtypes, termed adenosine A_1_, A_2A_, A_2B_ and A_3_ receptors, are widely distributed in human body and involved in several physio-pathological processes (Fredholm et al. 2001). The release of X-ray structures of the human A_2A_ AR in complex with agonists (Lebon et al. 2011, Xu et al. 2011) and antagonists (Jaakola et al.^2008^, Doré et al. 2011 Hino et al. 2012, Congreve, et al. 2012, Liu, et al. 2012) has enabled to extend structure-based drug design approaches to this class of receptors. With the use of homology modelling techniques, indeed, structural information on the other subtypes can also be derived. As a key step when building homology models is the selection of a proper template, we have developed a tool to guide the user in this crucial choice by implementing the “*Best Template Searching*” facility in our web-resource dedicated to ARs, *Adenosiland* (Floris et al. 2013). This tool is freely accessible at the following web address: http://mms.dsfarm.unipd.it/Adenosiland/ligand.php.

The underlying idea behind this facility is to help the user in selecting the best template or ARs model to get the highest quality receptor for further molecular docking studies. A possible strategy herein presented is to compute the similarity between a known or putative agonist/antagonist and all co-crystallized ARs ligands.

### Tool description

The “*Best Template Searching*” tool works as follows: the user is asked to input a query molecule either by uploading a SMILES string or by directly drawing the 2D structure by using the JME interface; the similarity of the input molecule is then computed against all the ligands co-crystallized with the hA_2A_ AR. The following similarity indexes are calculated: *(i)* shape similarity (based on the Manhattan distance between USR descriptors), *(ii)* 2D similarity (based on the Tanimoto and Tversky Similarities of Pubchem Fingerprints), *(iii)* pharmacophoric similarity (based on the Tanimoto similarity of Pharmacophoric triplets), and *(iv)* a combined similarity (derived by the following function: 0.6 * pharmacophoric similarity + 0.4 * shape similarity).

The values of the two coefficients composing the latter similarity index have been derived by running a preliminary in-house validation based on all available crystallographic structures: In particular, the two values have been chosen so that by providing as input the structures of the co-crystallized ligand the corresponding receptor structure results the best ranked one according to the combined similarity index. The values obtained for the structures considered for the internal validation are reported in Table 1. For all the structure except one, the suggested template results the corresponding crystal structure. The only exception is represented by NECA for which the structure co-crystallized with adenosine is suggested as best template. Considering the high structural similarity between the two agonist structures, the results is in line with the others.

**Table 1:** **Values of the in-house validation of the combined similarity index**

Input ligand	Suggested template	Combined similarity value
Adenosine	2YDO	0.83
NECA	2YDO	0.72
UK-432,097	3QAK	0.37
ZMA 241385	4EIY	0.69
T4G	3UZA	0.84
T4E	3UZC	0.92
XAC	3REY	0.67
Caffeine	3RFM	0.98

Simultaneously to the best template searching process, a similarity search screening is also performed against all adenosine agonists and antagonists deposited in ChEMBL, release 14 (Gaulton et al. 2011). In more details, the query is compared to 760 A_1_, 469 A_2A_, 559 A_2B_ and 290 A_3_ AR ligands and the comparison is based on the calculation of the similarity measures previously described. The identified compounds are reported in a table along with the associated binding data available in literature.

### Tool validation

**Figure 1:**
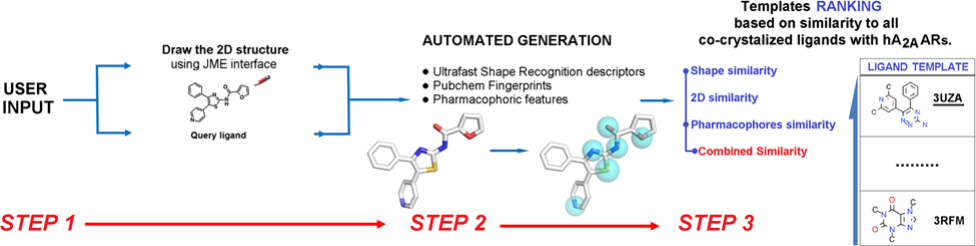
**Workflow of the homology modeling template selection based on the structure of furan-2-carboxylic acid (4-phenyl-5-pyridin-4-yl-thiazol-2-yl)-amide.**

Ligand similarity biased template selection criteria at the basis of the *“Best Template Searching”* tool has been successfully applied to rationalize the Structure Activity Relationships (SAR) of a series of [5-substituted-4-phenyl-1,3-thiazol-2-yl] furamides as antagonist of the hARs (Inamdar et al. 2013). The most potent derivative of the furamides series, the furan-2-carboxylic acid (4-phenyl-5-pyridin-4-yl-thiazol-2-yl)-amide, has been selected as query molecule: As reported in Table 2, a similarity sorting of the templates based on the combined similarity criteria has been taken into account to select the most suitable models for receptor-based ligand design. The selected workflow is summarized in Figure 1: Starting from the suggested best template, namely the structure with the 3UZA PDB ID, co-crystallized with the 6-(2,6-dimethylpyridin-4-yl)-5-phenyl-1,2,4-triazin-3-amine (T4G), we have constructed A_1_, A_2B_ and A_3_ AR models through homology modeling and used the so derived structural information to provide hypotheses of ligand-receptor interaction and ligand-receptor selectivity profile (Inamdar et al. 2013).

**Table 2:** **Similarity sorting of human A**_**2A**_ **AR templates based on furan-2-carboxylic acid (4-phenyl-5-pyridin-4-yl-thiazol-2-yl)-amide query ligand**

Ligand	PDB ID template	Shape similarity	2D similarity (Tanimoto)	2D similarity (Tversky)	Pharmacophore similarity (Tanimoto)	Pharmacophore similarity (Tversky)	Combined similarity (Shape & FP)
**T4G**	**3UZA**	**0.33**	**0.86**	**0.89**	**0.46**	**0.65**	**0.52**
ZM 241385	3PWH	0.58	0.90	0.93	0.27	0.42	0.48
T4E	3UZC	0.37	0.84	0.89	0.44	0.54	0.47
ZM 241385	4EIY	0.34	0.90	0.93	0.27	0.43	0.39
ZM 241385	3EML	0.35	0.90	0.93	0.27	0.42	0.39
NECA	2YDV	0.51	0.82	0.87	0.17	0.31	0.39
ZM 241385	3VG9	0.32	0.90	0.93	0.27	0.43	0.38
XAC	3REY	0.21	0.89	0.94	0.25	0.48	0.37
ZM 241385	3VGA	0.28	0.90	0.93	0.27	0.42	0.36
Adenosine	2YDO	0.33	0.82	0.86	0.18	0.31	0.31
Caffeine	3RFM	0.26	0.81	0.85	0.21	0.34	0.30
UK-432,097	3QAK	0.16	0.87	0.93	0.14	0.35	0.27

## Methods

The *“Best Template Searching”* tool is part of the Adenosiland infrastructure, based on Ubuntu 9.10 Linux operating system, which is a patchwork of several informatics tools (for more details see Floris et al. 2013). The similarity indexes are calculated by using different approaches: 2D similarity based on Tanimoto and Tversky indexes (Steinbeck et al. 2003,2006) are calculated from Pubchem Fingerprints (CDK implementation), the shape similarity is calculated by using an in-house implementation of the Ultrafast Shape Recognition method (Floris et al. 2011 Ballester and Richards 2007), and the pharmacophoric features of the pharmacophore-based similarity index are described by Gaussian 3D volumes (Taminau et al. 2008).

## Conclusions

We have implemented a novel tool, called “*Best Template Searching*” to provide template suggestions and homology models of all four hARs based on the similarity between a query structure provided by the user and all co-crystallized ARs ligands. It is well known that ligand-driven induced fit of the receptor is a key feature to facilitate the identification or the optimization of novel potent and selective agonists and antagonists, in particular through molecular docking studies. We therefore believe that choosing as template the structure co-crystallized with the ligand that shares the highest structural similarity with the scaffold of interest may represent an effective strategy. This is in facts the underlying idea of our platform implementation: By using the “*Best Template Searching*” option, users can upload a SMILES string or directly draw the 2D structure by using the JME interface of the scaffold of interest and search the most similar ligand co-crystallized so far with the hA_2A_ AR. Several similarity indexes are calculated by using different approaches such as a 2D similarity, shape similarity, pharmacophore-based similarity, and simple consensus shape- and pharmacophore-based similarity index.

We are also confident that the proposed strategy can be easily and effectively extended to other GPCRs.

## Abbreviations

ARs: Adenosine receptors
GPCRs: G protein-coupled receptors
NECA: N-ethyl-5′-carboxamido adenosine
T4E: 4-(3-amino-5-phenyl-1,2,4-triazin-6-yl)-2-chlorophenol
T4G: 6-(2,6-dimethylpyridin-4-yl)-5-phenyl-1,2,4-triazin-3-amine
ZM 241385: 4-(2-(7-amino-2-(2-furyl)(1,2,4)triazolo(2,3-*a*)(1,3,5)triazin-5-yl-amino)ethyl)phenol
XAC: N-(2-aminoethyl)-2-[4-(2,6-dioxo-1,3-dipropyl- 2,3,6,7-tetrahydro-1H-purin-8-yl)phenoxy]acetamide.
